# Conversion of KEGG metabolic pathways to SBGN maps including automatic layout

**DOI:** 10.1186/1471-2105-14-250

**Published:** 2013-08-16

**Authors:** Tobias Czauderna, Michael Wybrow, Kim Marriott, Falk Schreiber

**Affiliations:** 1 Leibniz Institute of Plant Genetics and Crop Plant Research (IPK), Gatersleben, Germany; 2 Caulfield School of Information Technology, Monash University, Victoria 3145, Australia; 3 Institute of Computer Science, Martin Luther University Halle‐Wittenberg, Halle (Saale), Germany

## Abstract

**Background:**

Biologists make frequent use of databases containing large and complex biological networks. One popular database is the Kyoto Encyclopedia of Genes and Genomes (KEGG) which uses its own graphical representation and manual layout for pathways. While some general drawing conventions exist for biological networks, arbitrary graphical representations are very common. Recently, a new standard has been established for displaying biological processes, the Systems Biology Graphical Notation (SBGN), which aims to unify the look of such maps. Ideally, online repositories such as KEGG would automatically provide networks in a variety of notations including SBGN. Unfortunately, this is non‐trivial, since converting between notations may add, remove or otherwise alter map elements so that the existing layout cannot be simply reused.

**Results:**

Here we describe a methodology for automatic translation of KEGG metabolic pathways into the SBGN format. We infer important properties of the KEGG layout and treat these as layout constraints that are maintained during the conversion to SBGN maps.

**Conclusions:**

This allows for the drawing and layout conventions of SBGN to be followed while creating maps that are still recognizably the original KEGG pathways. This article details the steps in this process and provides examples of the final result.

## Background

### Biological network sources

Life scientists commonly use biological networks from online databases in research and teaching. As an example, metabolic pathways are of high interest for exploring organism‐specific metabolism, mapping ‐omics data onto metabolic networks for further analysis, and simulating metabolic processes using techniques such as flux balance analysis.

There are various online repositories for biological pathways, see http://www.pathguide.org/. Here we will concentrate on metabolic networks. Major databases for metabolism are the Kyoto Encyclopedia of Genes and Genomes (KEGG) PATHWAY [1], a multi‐organism pathway database containing thousands of metabolic pathways, represented as manually drawn pathway maps; BioCyc/MetaCyc [2], a collection of organism‐specific pathway databases; Reactome [3], a multi‐organism pathway database initially established with a focus on human biology; and PANTHER pathway [4], also a multi‐organism pathway database. There are also many special metabolic pathway databases covering a specific species or group of species, e. g., PlantCyc [5] and MetaCrop [6] for plants.

KEGG provides graphical representations for pathways and the layout information is publicly available for download via the XML‐based KGML file format. As KEGG also contains the largest collection of metabolic pathways we choose this database for our work.

### Biological network visualization

Biological network visualization requires (1) single biological elements to be represented by meaningful graphical symbols (*glyphs*), and (2) the spatial placement (*layout*) of these glyphs to form a readable map.

**Figure 1:**
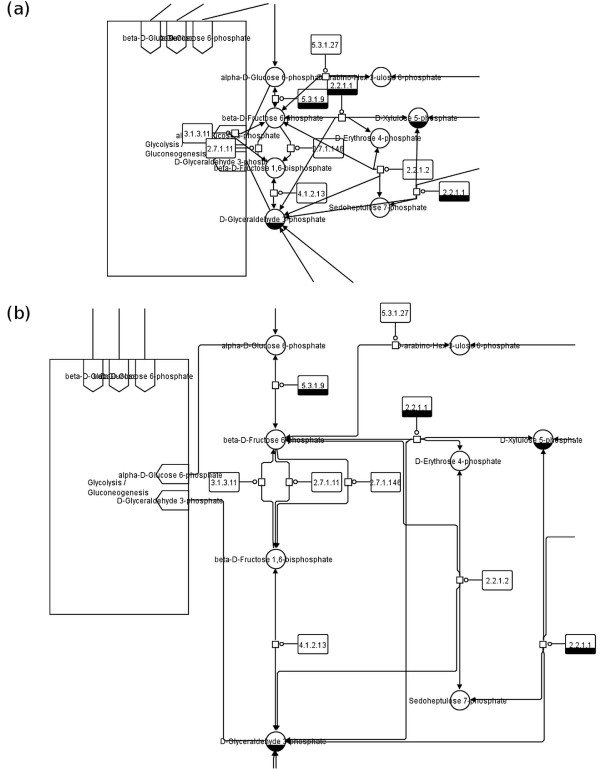
**Pentose phosphate pathway from the KEGG database.** Part of the pathway map shown as **(a)** SBGN representation after translation without layout adjustment, and **(b)** SBGN representation after translation with constraint layout applied (see also Figures 2, 3 and 4 for a complete version of the map).

Exchange of information between humans can often be enhanced by the use of well‐defined unambiguous standards for visual representation. While informal drawing conventions exist for the visualization of biological networks, arbitrary graphical representations are still commonly used. Uniform systems of nomenclature describing the components of networks based on a well‐defined set of symbols are well established within fields such as engineering, computer science, and physics. For the visual representation of biological networks and cellular processes the Systems Biology Graphical Notation (SBGN) has been recently introduced [7]. Similar to wiring maps in electrical engineering, SBGN allows the unambiguous representation of biological knowledge using a limited number of easily recognizable glyphs. The three different languages (Process Description, Entity Relationship, Activity Flow) covered by SBGN enable the representation of any kind of biological network such as metabolic, regulatory, and interaction networks on different levels of granularity. Figure 1(b) shows an example SBGN Process Description map.

**Figure 2:**
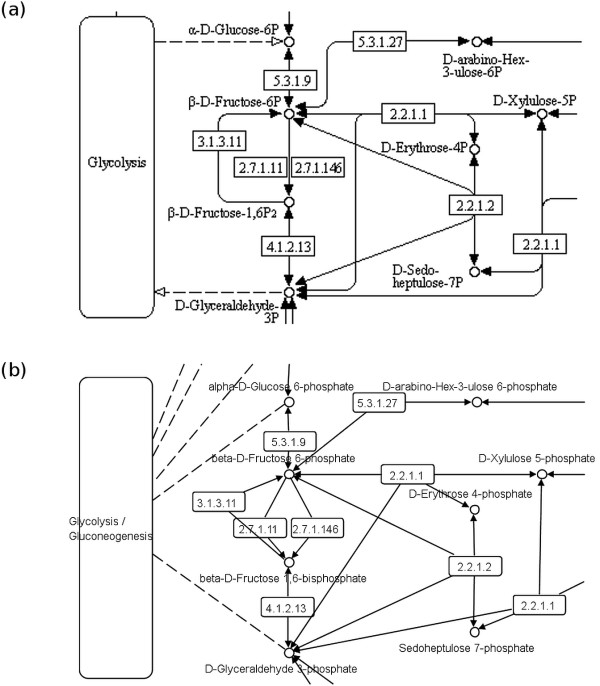
**Pentose phosphate pathway from the KEGG database.** Part of the pathway map shown as **(a)** KEGG reference image, and **(b)** KGML representation (see also Figure 1).

**Figure 3:**
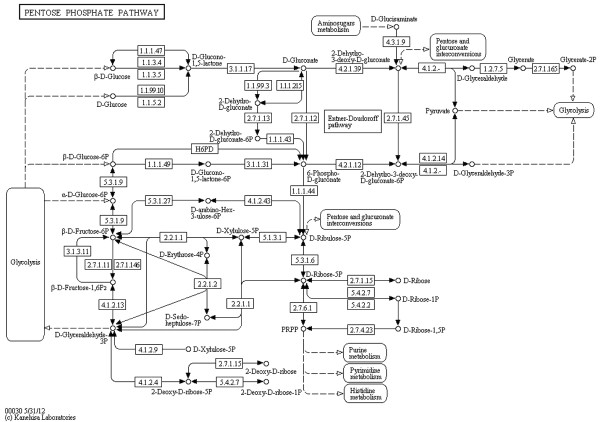
**Pentose phosphate pathway (KEGG).** Pentose phosphate pathway from the KEGG database (http://www.kegg.jp/kegg/pathway/map/map00030.html), reference image.

**Figure 4:**
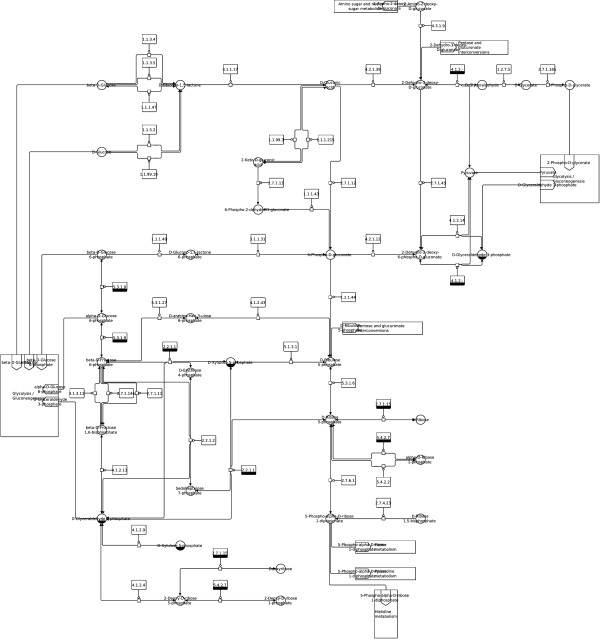
**Pentose phosphate pathway (SBGN).** Pentose phosphate pathway from the KEGG database (http://www.kegg.jp/kegg/pathway/map/map00030.html), downloaded as KGML file, translated to SBGN and laid out using constraints with the described method.

Ideally, online repositories for biological networks would provide networks in a variety of notations including SBGN, automatically converting between them as necessary. Such conversions should preserve the existing layout as much as possible, since it will often have been carefully chosen by a human expert to emphasize important biological features in the map. Unfortunately, this is non‐trivial because the conversion may add, remove or alter map elements, preventing use of the exact same layout.

Automatic layout of biological networks can be done with graph drawing algorithms, see the book of [8]. These techniques have also been applied to specific biological network layout applications, such as for signal transduction maps (e. g., [9]), protein interaction networks (e. g., [10]), and metabolic pathways (e. g., [11,12]). However, manually drawn layouts tend to be easier to understand and aesthetically preferable to automatic layouts. Furthermore, automatic layout methods often cannot fulfill particular specific layout requirements, such as those given in the SBGN specification. For example, these approaches do not allow specification of positions of enzymes or modifiers relative to processes, layout for reaction groups, or specific bundled routing via certain paths to draw visual attention to particular structures.

Here we describe a method to automatically translate the widely used KEGG metabolic pathways into SBGN format. We infer important properties of the KEGG layout and model these as layout constraints that are maintained during the conversion to SBGN. This allows for style and layout conventions of SBGN to be followed while creating maps that are still recognizably the same pathway.

Our approach (see also Figures 1 and 2) relies on (1) using SBGN as an unambiguous graphical representation for biological objects and interactions, and (2) solving geometric constraints which capture structural layout requirements (e. g., non‐overlap of map elements) as well as arrangement preferences of the original author (e. g., alignment) for automatic layout based on the original KEGG map.

There are three main steps. The first step is to convert the KEGG map into the SBGN Process Description (PD) [13] notation, adding and deleting nodes and edges where necessary. The second step involves finding an arrangement for the nodes in the diagram. It is based on the constraint‐based layout method in [14] which allows a network map to be laid out subject to computed or user‐specified geometric placement constraints, such as non‐overlap, alignment, and containment. In this step we start with the layout of the initial KEGG maps to infer important structural constraints, such as relative orderings and alignment. We remove overlap between new or modified nodes while enforcing containment relationships, preserving structural constraints and following the layout guidelines of SBGN. In the third step, we perform orthogonal edge routing to create routes for edges which do not overlap nodes or each other [15]. Our basic approach of using inferred constraints to preserve existing layout while specifying further constraints to enforce required drawing conventions is a powerful and flexible technique which could easily be adapted for translating between other biological network notations.

Constraint‐based layout techniques originate with the early CAD tool SketchPad [16] and are now widely used in GUI widget layout, CAD systems and diagramming tools. They have been used for a variety of purposes: to support parametric objects whose shape changes to different design contexts, automatic adjustment of user interfaces and maps to different viewing contexts (e. g., [17]), enforcing similarity between consecutive layouts in interactive and other dynamic settings (e. g., [18]), preserving geometric relationships during editing (e. g., [16]), and tailoring network map layouts to take into account layout styles and user interests (e. g., [19]). To the best of our knowledge our use of them to preserve the user’s “mental map” of a layout during translation between two different map notations is novel.

Geometric constraints can either be inferred from a map or explicitly imposed by the user. Typically constraint inference is based on map elements satisfying a possible constraint within some error tolerance [20,21], but may also take into account syntactic requirements of the particular map notation [22]. A wide variety of different techniques have been suggested for solving geometric constraints in graphical applications [23]. Our approach utilizes constrained‐satisfaction methods for constrained graph layout [14,19] in combination with automatic orthogonal edge routing techniques [15].

Some of the closest work to ours is research on converting from SBML [24] or BioPAX [25] formats into graphical formats like SBGN. Some examples for SBML are Arcadia [26], which uses GraphViz [27] for layout and the SBML Layout Extension [28]. BioPAX to SBGN conversion is done, for example, by Paxtools [29] and BioUML [30]. However, these approaches can’t translate the widely used KEGG maps into SBGN, and they mostly compute entirely new layouts rather than utilizing layout information derived from the original (KEGG, SBML, or BioPAX) map. Tools such as KEGGtranslator [31] and MGV [32] can load and translate KEGG maps but also do not support layout adjustment based on information from the original KEGG map.

## Methods

### Translation of KEGG to SBGN

Some pathway database providers have begun to adopt SBGN for the graphical representation of pathways [3,4,6,33,34]. However, the popular KEGG database still provides pathway maps in its own representation both as static image files and as KGML files for utilization in software tools.

The KGML files serve as the basis for our translation from KEGG to SBGN since they contain all information (including layout information for pathway entities) necessary to reconstruct a pathway map. Note that the static KEGG images contain often less edges than the corresponding KGML file as several reactions are often manually reduced to a single reaction in the static image. We will focus on the translation of metabolic pathways from the KEGG representation (KGML) to SBGN Process Description (PD) maps.

In general, a pathway map can be considered as a graph *G*=(*V*,*E*) composed of a set of nodes *V* representing pathway entities and a set of edges *E*, where each edge connects two nodes and thus represents a relation between the pathway entities. KEGG pathway maps in particular consist of several types of nodes and edges [35]^a^. Let *G*_*K*_=(*V*_*K*_,*E*_*K*_) be a graph representing a metabolic pathway map from the KEGG database. In metabolic pathway maps from KEGG all three types of nodes can be found: (1) *gene product, mostly protein but including RNA* node *v*_*GP*_∈*V*_*K*_, (2) *other molecule, mostly chemical compound* node *v*_*OM*_∈*V*_*K*_, and (3) *another map* node *v*_*AM*_∈*V*_*K*_. In addition, two types of edges can be found: (1) *molecular interaction or relation* edge *e*_*MI*_∈*E*_*K*_ and (2) *link to another map* edge *e*_*LM*_∈*E*_*K*_. A typical drawing of a KEGG pathway map can be seen in Figure 2(a), a *gene product* node *v*_*GP*_ is drawn as a rectangle showing a reaction, an *other molecule* node *v*_*OM*_ is represented by a circle showing a substrate or product of a reaction, *another map* node *v*_*AM*_ is drawn as a rectangle with rounded corners, a *molecular interaction or relation* edge *e*_*MI*_ is shown as an edge with a filled arrowhead, and a *link to another map* edge *e*_*LM*_ is drawn as a dotted edge with an empty arrowhead.

For all elements of a KEGG metabolic pathway map a respective element (or several respective elements) in the SBGN Process Description (PD) language can be found; for a description of all SBGN PD elements see [13]. However, to create a valid SBGN PD map the number of nodes and edges increases during the translation as described below. Let *G*_*S*_=(*V*_*S*_,*E*_*S*_) be a graph representing the SBGN PD map. The translation of an *other molecule* node, *another map* node, and a *link to another map* edge from KEGG to SBGN PD is a one‐to‐one translation: an *other molecule* node *v*_*OM*_ is mapped to a *simple chemical* node *v*_*SC*_∈*V*_*S*_, a *another map* node *v*_*AM*_ is mapped to a *submap* node *v*_*SM*_∈*V*_*S*_ including the required number of *terminal* nodes *v*_*TE*_∈*V*_*S*_, and a *link to another map* edge *e*_*LM*_ is mapped to an *equivalence arc* edge *e*_*EA*_∈*E*_*S*_. A reaction in a KEGG pathway map shown by a *gene product* node *v*_*GP*_ is translated to a *macromolecule* node *v*_*MA*_∈*V*_*S*_ showing the enzyme catalyzing the reaction plus an additional *process* node *v*_*PN*_∈*V*_*S*_ showing the reaction itself. Both nodes are connected by an additional *catalysis arc* edge *e*_*CA*_∈*E*_*S*_. The translation of a reaction node therefore increases the number of nodes and edges. *Molecular interaction or relation* edges *e*_*MI*_ have to be translated according to the reaction they are connected to. In case of an irreversible reaction the edge *e*_*MI*_ from the substrate node to the reaction node is translated to a *consumption arc* edge *e*_*CA*_∈*E*_*S*_ and the edge *e*_*MI*_ from the reaction node to the product node is translated to a *production arc* edge *e*_*PA*_∈*E*_*S*_. For reversible reactions the translation is simplified, all edges *e*_*MI*_ are translated to *production arc* edges *e*_*PA*_∈*E*_*S*_ indicating the reversibility of the reaction. See Table 1 for more information about the translation process, Figures 1 and 2 for detailed examples and Figures 3 and 4 for the full maps.

**Table 1:** Translation of KEGG metabolic pathways to SBGN PD maps using KGML files

	**KEGG representation**	**KGML representation**	**SBGN PD representation**
Irreversible reaction			
Reversible reaction			
Several reactions between compounds			
Link to another map			

The KEGG, KGML, and SBGN PD representation is shown for irreversible reaction, reversible reaction, several reactions between compounds, and link to another map.

As an added complication, the KGML files for some pathway maps have errors and do not contain the complete information necessary for a correct automatic translation. Typical KGML errors are missing information about (1) the reversibility of a reaction, (2) the type of a compound (substrate or product) or (3) substrates and products of a reaction at all. We automatically detect these rare cases and render the relevant nodes and edges in red to highlight the ambiguity.

### Layout process

Pathway diagrams contained in the KEGG database are manually drawn [35] and provide a layout that emphasizes the biological features regarded as important by the author. A reference image for each pathway is available online (http://www.kegg.jp/kegg/pathway.html). These images show the pathways in a simplified manner where the number of edges is reduced. In principle, a reaction taking place between two compounds is shown by an edge drawn from the substance to the reaction node and an edge drawn from the reaction node to the product. If several reactions take place between two compounds there is only one edge drawn between the two compounds and the reaction nodes are drawn together underneath or above the edge.

In contrast, the KGML files provided by the KEGG database contain enough information to reconstruct all nodes and all edges but do not include the complete layout information. The files only contain node layout information (node positions, node sizes) but do not contain edge routing information. Thus, a KEGG pathway map can be reconstructed from a KGML file that preserve the author’s intended layout except for the edge routing (see Figure 2(b)).

The translation of KEGG metabolic pathways to SBGN PD maps increases the number of nodes and edges as described in Section “ Translation of KEGG to SBGN ” and thereby breaks the initial layout by introducing node overlaps (see Figure 1(a)).

Scaling the space between nodes in both the *x* and *y* dimension would be a straightforward solution to eliminate node overlaps but has the disadvantage that the maps become unnecessarily large due to increased whitespace. Furthermore, we would still need to produce new routes for edges. For this reason we perform automatic layout of the SBGN PD maps. This process is designed to (1) preserve the original layout intent of the author, including the use of orthogonal edge routing and (2) arrange the pathway map according to SBGN layout rules.

The specification for SBGN PD maps describes requirements and recommendations for layout which govern the visual appearance and aesthetics of the Process Description (PD) language [13]. Layout requirements and recommendations most important for the translation of KEGG metabolic pathway maps to SBGN PD maps are summarized below: 

1. nodes are not allowed to overlap, except where one node is contained by another;

2. if an edge crosses a node it must be drawn on top, it is recommended that edges not cross nodes;

3. edges are not allowed to overlap the border line of nodes;

4. edges are not allowed to overlap each other (only crossing is allowed);

5. consumption and production arcs are attached to the center of opposite sides of a process node;

6. catalysis (modulatory) arcs are attached to the two other sides of the process node;

7. at least a part of a label has to be placed inside the node it belongs to;

8. labels should be horizontal; and

9. the number of crossings between edges should be minimized.

Conceptually, the layout process is performed in two main steps. The first stage determines a position for the nodes in the SBGN map. It starts with a desired position for each node based on their positions in the KEGG map and a set of geometric constraints. Then a greedy heuristic is used to find node positions that satisfy these constraints and avoid overlap between nodes (Requirement 1 above). The second stage is to take the resulting node positions as well as edge connection information and compute orthogonal object‐avoiding paths for edges. The paths satisfy Requirements 2–6. In the next sections we describe these stages in more detail.

We follow Requirements 7 and 8 when drawing labels, although it is often unavoidable that this results in text that overlaps other objects. The final recommendation of minimizing edge crossings is a known intractable problem [36], but we employ heuristic approaches that give reasonable results.

#### Computing node positions

**Figure 5:**
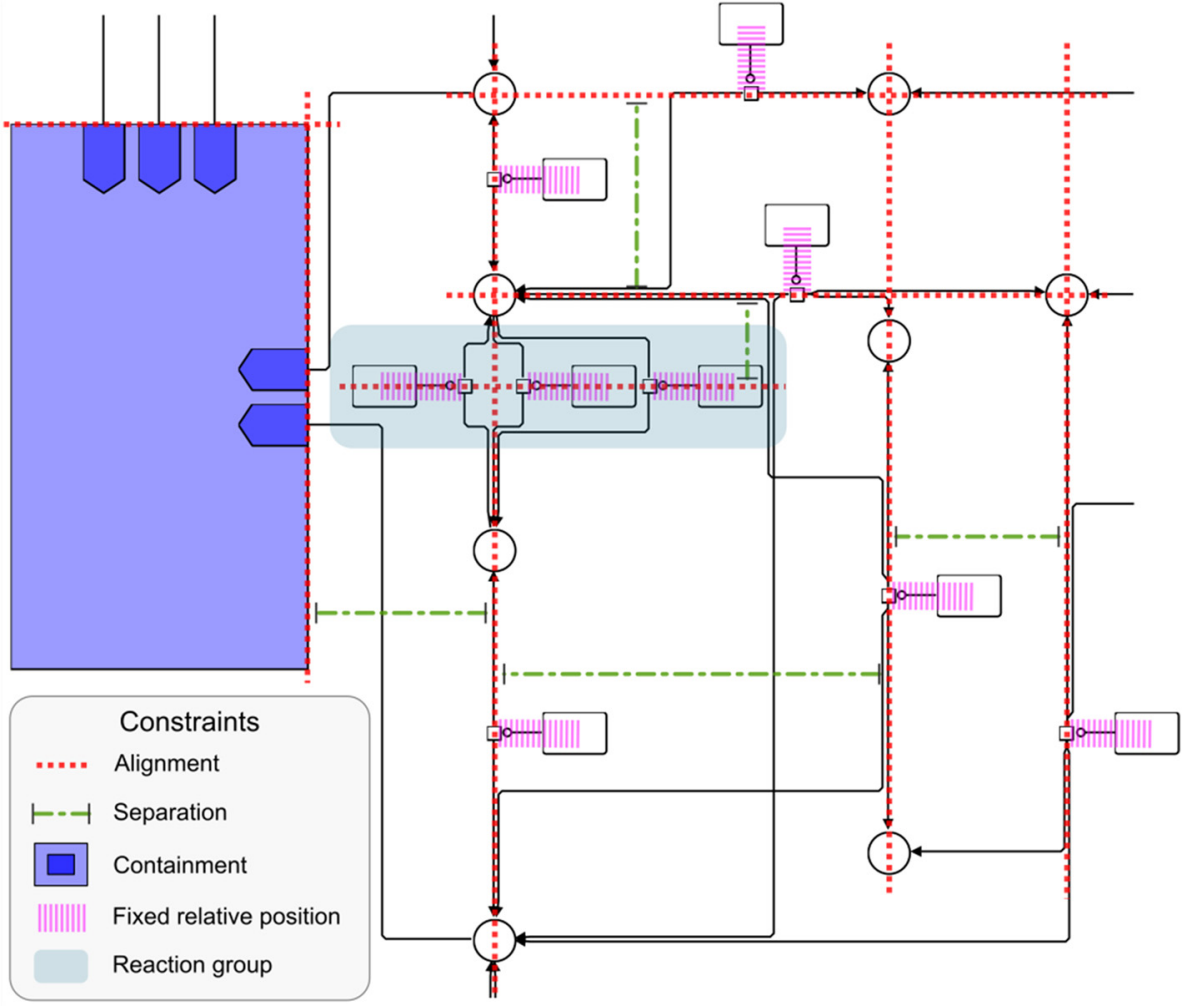
**Pentose phosphate pathway from Figure 1(b).** Pentose phosphate pathway from Figure 1(b) with alignment, separation, containment, fixed‐relative‐position constraints and reaction group shown. Non‐overlap constraints are determined automatically, taking into account containment constraints. Node labels have been removed for better readability.

The first stage of the layout process is to find node positions using constraint‐based layout. This takes sizes and a desired position for each node (from the KGML layout description file) as well as a set of constraint relationships that we would like to be satisfied. These constraints are of three types: (1) **Recognizability:** Alignment and separation constraints are used to preserve recognizability of the original KEGG layout and the author’s original layout intent; (2) **Beautification:** Non‐overlap and spacing constraints are used to make sure the new layout is not bad; and (3) **Style:** Enforcement of SBGN style using containment and alignment constraints for hierarchical nodes and fixed‐relative‐position constraints to keep macromolecules positioned in relation to process nodes in a reaction and non‐overlap of nodes. These constraints are shown for our previous example in Figure 5.

Recognizability constraints are determined by analyzing the network to find groups of nodes that are visually aligned in the original KEGG layout. When looking for *x* or *y* alignment we use a small tolerance to include cases where there was an obvious visual intention to align objects but they are not pixel perfectly aligned. This alignment inference is performed for all compounds (simple chemicals in SBGN) and reactions (process nodes in SBGN) regardless of whether or not they are connected. Once we have determined the alignment relationships we add separation constraints between each alignment group to preserve the relative orthogonal order of these within the layout. If two compounds are aligned either in *x* or *y* direction and several reactions take place between them, additional alignment and separation constraints are defined for these reactions to line them up in horizontal or vertical *reaction groups* (see Figure 5).

Beautification constraints are non‐overlap constraints generated between all nodes to stop them from overlapping and to leave enough empty space between them for subsequent edge routing. To achieve this spacing, we specify slightly enlarged sizes for nodes during the layout step.

In order to satisfy the drawing conventions of SBGN we define Style constraints for the containment of hierarchical nodes and to keep particular nodes in a fixed position relative to each other.

According to the SBGN specification, submaps contain terminal nodes graphically shown as overlapping nodes contained within them but sharing a border. Thus containment constraints have to be defined for submaps and the corresponding terminals to force them to be positioned within their parent node and to prevent overlap constraints being generated between them. Additionally, an alignment constraint between a submap and each of its terminals has to be defined to keep the terminals positioned on the border of the submap.

For a macromolecule and a process node connected by a catalysis arc the relative position is fixed by two constraints, either an alignment constraint in *x* direction and a separation constraint specifying a fixed distance in *y* direction or vice versa.

For the layout, the high‐level geometric relationships we use are represented at a low level in the solver as multiple *separation constraints* of the form *u*+*g*≤(=) *v*, enforcing a minimum (or precise) gap *g* between the positions *u* and *v* of pairs of objects in either the *x* or *y* dimensions of the drawing [19]. For example, a vertical alignment between three nodes would be represented as a position variable for the alignment and three equality constraints that force the *x* position of each node to be the same as the alignment position. Alignment, separation and fixed‐relative‐position constraints are specified at this high‐level. We use the algorithm from [19] to *project* the desired position of the objects onto the low level separation constraints—this means that objects are placed as close as possible to the desired position while satisfying the layout constraints.

However, not all layout constraints have a direct translation to separation constraints. For instance, non‐overlap of two objects can be modeled by choosing to constrain the first object to be “above”, “below”, “left” or “right” of the second: the best choice depends upon the desired object position and interaction with other constraints. We solve this by using the greedy heuristic described in [14]. Using this method, we do not specify non‐overlap constraints using individual separation constraints, but instead let the solver compute the choice for how best to resolve overlap. Importantly, we give non‐overlap constraints a lower priority, hence considering them after all other constraints have been satisfied. The four alternatives for enforcing each non‐overlap constraint are ranked based on minimizing potential node movement. We try adding each alternative in turn until we find one that does not cause itself or previously added constraints to become unsatisfied. Each time we encounter an unsuccessful alternative we backtrack by resetting node positions to their earlier values and try a different alternative.

Since we require the non‐overlap requirement to be relaxed for nodes contained within submaps, we extended the method in [14] so that containment hierarchies may be specified. The solver then considers these containment relationships when resolving non‐overlap, and instead generates separation constraints keeping children inside parent nodes as well as non‐overlap constraints between siblings at each level of the hierarchy. An added benefit of this is that nodes at each level of the hierarchy are standard objects within the layout engine, which allows additional constraints between them, such as the alignment of terminal nodes on the boundary of submaps.

#### Edge routing

To achieve high quality layout that follows the drawing conventions of SBGN there are several requirements for the edge routing. We require that edges do not cross or touch nodes other than at the point where they are connected to that node. The edge routes themselves should be orthogonal paths made up of only vertical and horizontal line segments with the minimum cost (i. e., weighted sum of the total length and number of segments). We draw these edges with slight rounded corners but this is not a consideration in the routing problem itself. Finally, where resultant edge routes share paths with each other, even when connected to a common node, we require that they are nudged apart so that they may be visually discerned, as per the SBGN specification.

Edge routing is performed using a technique that generates an orthogonal visibility graph from a set of rectangular obstacles and uses this to generate orthogonal object‐avoiding routes [15]. The edge router requires us to give the locations of all nodes as rectangles with positions, as well as the edges and information on the nodes to which they are connected. We are able to specify connection ports on a node, which are specific positions and directions by which an edge must be routed into the node.

For most nodes we require that edges connect from any direction to the center of the node, though are only drawn to its border. For process nodes and macromolecules we define specific ports on the nodes which specify a particular position and direction that catalysis, consumption and production arcs must attach to. We use expanded node dimensions for process nodes and macromolecules, so that no edges cross catalysis arcs. We also specify connection ports on terminal nodes to force edges to connect to them at the boundary they share with their containing submap.

For reaction groups we route edges from each of the two compounds together so they diverge at a common point inside the reaction group, see Figure 5. This was done by extending the routing method in [15] to allow specification of *checkpoints* that must be visited by a route. This effectively involves routing individual sub‐routes between pairs of endpoints or checkpoints along a route while appropriately penalizing bends occurring at the checkpoints.

After routing is performed, we use the nudging feature of [15] to separate overlapping edge routes. We extended this nudging to also include final edge segments attached to nodes. While these would usually be fixed in place due to attaching to a pin or the center of a node, if multiple edges all enter a node from one side we allow these to be spaced apart up to the bounds of the node. We also modified the nudging to take checkpoints into account.

We also found that it was fairly common for fixed‐distance nudging to fail when there were many edges running through a limited space. For this reason we made the fairly simple extension of having the router recursively try increasingly smaller nudging distances for individual bundles of edges when this occurs.

## Results and discussion

Our implementation of the described technique for translation of KEGG pathways into SBGN is available in SBGN‐ED [37], an add‐on for the VANTED framework [38]^b^. This article describes a complete automated process for translating KEGG maps to SBGN, including detection and display of ambiguous information^c^. In addition to the translation process, we detail additions to our previous constraint‐based layout [14] to allow containment hierarchies. The article also extends our prior edge routing work [15] with checkpoints and improved nudging, which permit edge bundling for reaction groups.

**Figure 6:**
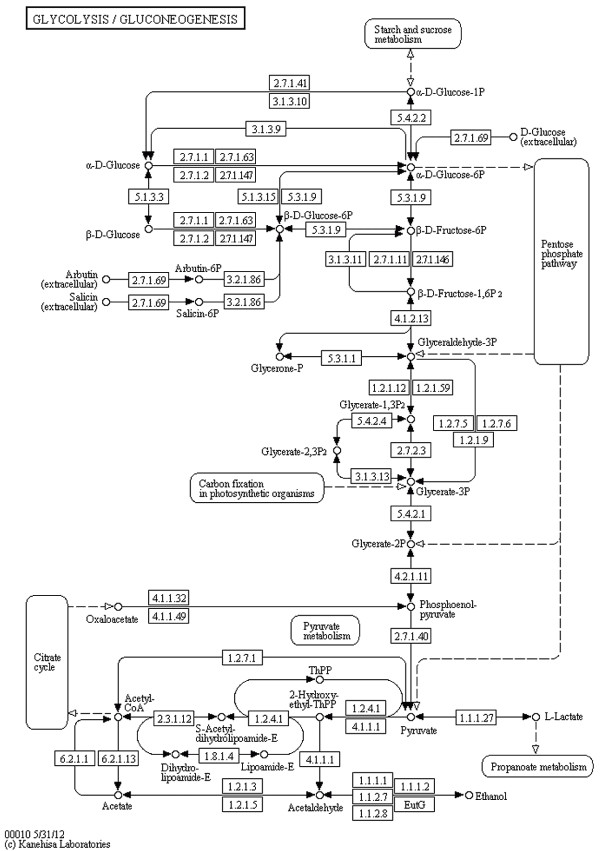
**Glycolysis / Gluconeogenesis (KEGG).** Glycolysis / Gluconeogenesis from the KEGG database (http://www.kegg.jp/kegg/pathway/map/map00010.html), reference image.

**Figure 7:**
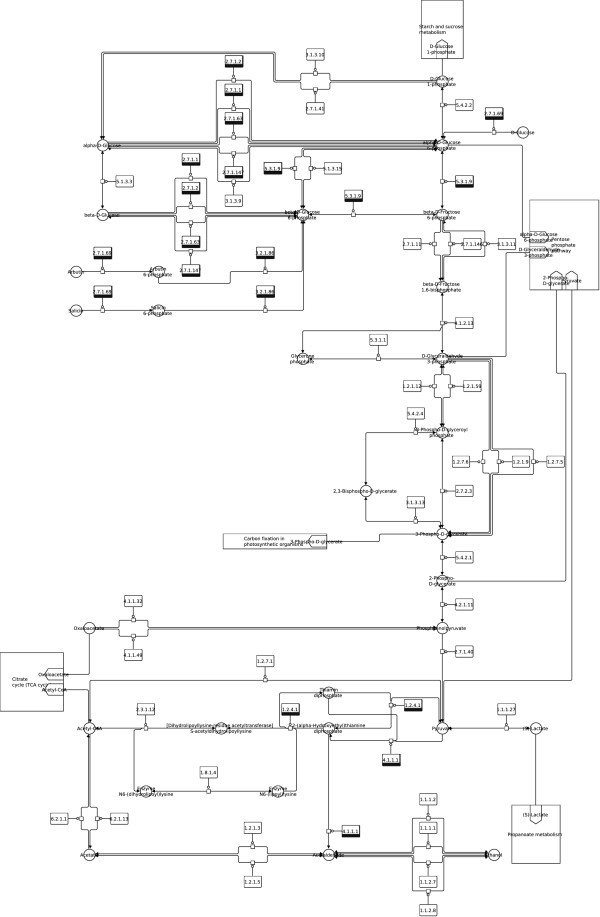
**Glycolysis / Gluconeogenesis (SBGN).** Glycolysis / Gluconeogenesis from the KEGG database (http://www.kegg.jp/kegg/pathway/map/map00010.html), downloaded as KGML file, translated to SBGN and laid out using constraints with the described method.

**Figure 8:**
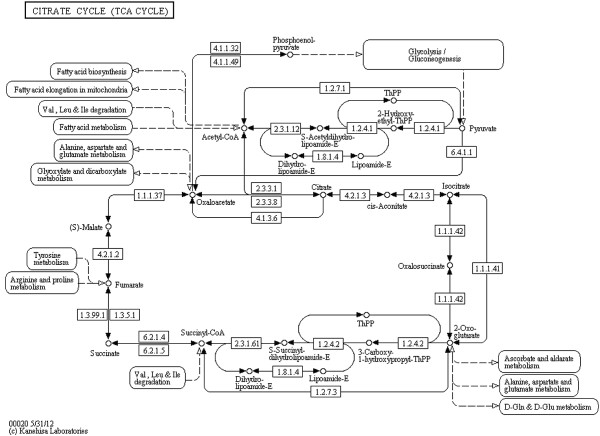
**Citrate cycle (TCA cycle) (KEGG).** Citrate cycle (TCA cycle) from the KEGG database (http://www.kegg.jp/kegg/pathway/map/map00020.html), reference image.

**Figure 9:**
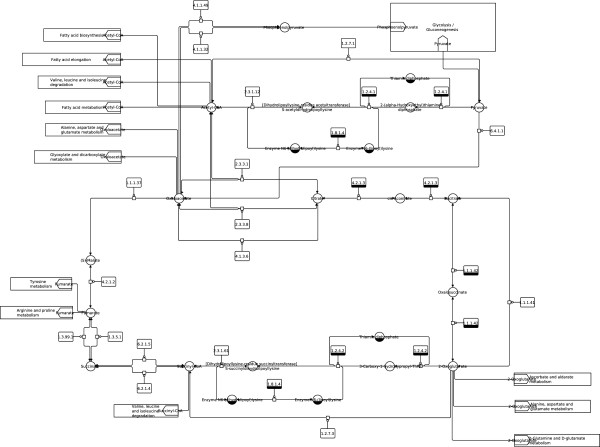
**Citrate cycle (TCA cycle) (SBGN).** Citrate cycle (TCA cycle) from the KEGG database (http://www.kegg.jp/kegg/pathway/map/map00020.html), downloaded as KGML file, translated to SBGN and laid out using constraints with the described method.

**Figure 10:**
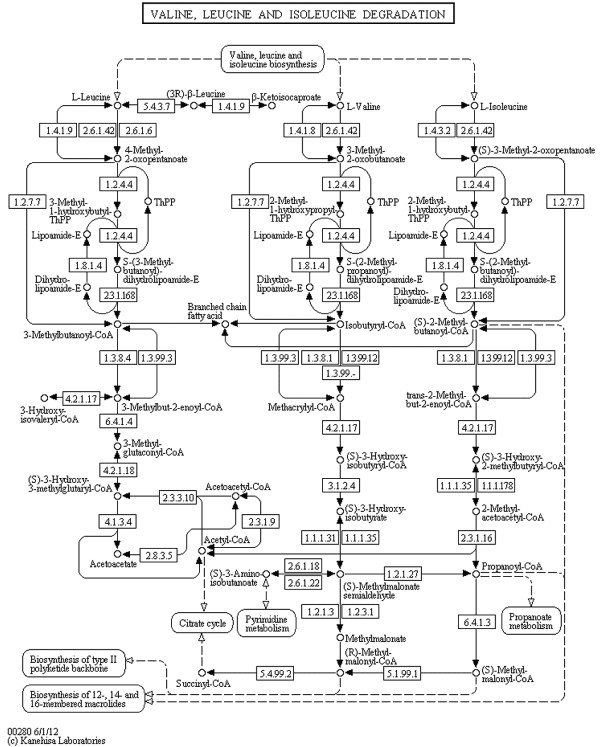
**Valine, leucine and isoleucine degradation (KEGG).** Valine, leucine and isoleucine degradation from the KEGG database (http://www.kegg.jp/kegg/pathway/map/ map00280.html), reference image.

**Figure 11:**
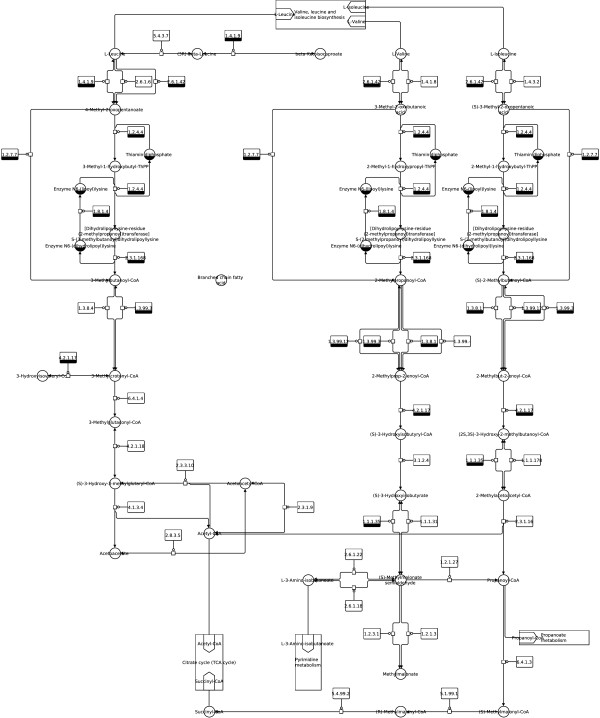
**Valine, leucine and isoleucine degradation (SBGN).** Valine, leucine and isoleucine degradation from the KEGG database (http://www.kegg.jp/kegg/pathway/map/ map00280.html), downloaded as KGML file, translated to SBGN and laid out using constraints with the described method.

We have applied this completely automated process to a large number of examples from the KEGG database. Some examples of the SBGN diagrams along with the original KEGG reference images are shown in Figures 3, 4, 6, 7, 8, 9, 10 and 11. It takes an average of four seconds to complete the translation and layout process for an individual pathway. For some larger pathway maps from the KEGG database with up to 300 nodes the overall process can take up to 15 seconds.

Notice that layout features and the overall look of the original maps are retained in the produced SBGN examples, including prominent vertical pathways in Figures 6, 7, 10 and 11, the TCA cycle in the lower half of Figures 8 and 9, and recognizable reaction loops in all three of these examples. The quality of our produced layouts show that the described method does a good job of translating KEGG maps into SBGN maps while preserving important aspects of the layout.

There are also some limitations to our approach. Firstly, it can be slow for very large examples. While it is fast for small examples and the layout algorithms have polynomial complexity, they can take up to 30 minutes for a network with 2,000 nodes and 2,000 edges. We are investigating some possible improvements in this area. Secondly, we currently do not focus on producing compact layouts, though it would be possible to adapt the techniques to achieve this and still satisfy all the layout constraints. Thirdly, we can sometimes have issues positioning large numbers of edges routed along common paths if there is not enough space between elements for them to be ideally spaced. We could look at moving objects slightly to make space in this case, again taking advantage of the layout constraints so as not to degrade the quality of the layout.

While not necessarily a limitation of our approach, labels on the maps we produce can be difficult to read when they overlap with other elements. The SBGN specification requires that shapes representing compounds have a fixed shape, rather than being sized to fit labels. Also, it dictates that labels must be drawn on shapes, which precludes us employing various approaches to map labeling that have been investigated to solve this general problem. The SBGN working group are aware of the problem and these requirements will hopefully be changed in future revisions of SBGN.

## Conclusions

Databases of biological networks are widely used in research and teaching by life scientists. While graphical representation of these networks follow some common drawing conventions they still often make use of various somewhat arbitrary notations. Ideally, databases containing biological networks should provide these networks in a variety of graphical representations including SBGN.

We have described a method to automatically translate pathway maps from the well‐known and widely used KEGG pathway database into a SBGN representation. We employ a constraint‐based layout method to follow drawing and layout conventions of SBGN while preserving important layout features of the KEGG layout, allowing the resulting map be easily read and to remain recognizable as the original. The latter is especially important since KEGG pathways are manually drawn so that their layout emphasizes biological relationships regarded as important by domain experts.

Our proposed constraint‐based layout method could be adapted for use on SBGN maps converted from SBML or BioPAX formats. Similarly, these maps would mainly consist of the SBGN elements *simple chemical*, *macromolecule*, *process*, and the corresponding arcs. The main difference is that position information is not specified in BioPAX format or in SBML format (when not extended by layout information using the SBML Layout Extension [28]). Thus positions have to be determined from the context of the map, e. g., fixed relative position of a macromolecule and a process node connected by a modulatory arc, or positioned first with more traditional graph layout approaches.

In terms of future work, we would like to investigate doing some form of compaction on the final layout, since our method can still sometimes result in unnecessary white‐space being added into the maps. We would also like to improve the case of detecting otherwise unconstrained reactions that appear to be incorrectly left out of alignment relationships. Adjusting the alignment inference tolerance may solve this for individual maps, but we would like to do this analysis by looking at the network structure as well as the inferred constraints so that it works more generally. While not particularly difficult, solving these issue are important since they tend to be examples of the more obvious problems that users will notice in automatically generated layouts. It could also be interesting to translate the large KEGG global maps into SBGN using our technique.

## Availability and requirements

•**Project name:** SBGN‐ED

•**Project home page:**http://www.sbgn‐ed.org

•**Operating system(s):** Windows (32‐bit, 64‐bit), Linux (32‐bit, 64‐bit), and Mac OS (32‐bit, 64‐bit)

•**Programming language:** Java 6/7

•**License:** GNU GPL 2.0

## Endnotes

^a^ For a detailed description see http://www.kegg.jp/kegg/document/{help}_pathway.html.

^b^ SBGN‐ED with KEGG to SBGN translation including automatic layout is currently available for Windows (32‐bit, 64‐bit), Linux (32‐bit, 64‐bit), and Mac (32‐bit, 64‐bit).

^c^ In contrast, our previous simple KEGG‐SBGN conversion in [37] did not translate all elements (such as groups), did not highlight errors from the KGML, and included no layout or edge routing other than scaling up the entire diagram to reduce overlaps.

## Competing interests

The authors declare that they have no competing interests.

## Authors’ contributions

TC implemented the KEGG to SBGN translation, integrated the layout algorithms into SBGN‐ED and wrote the code to infer and specify constraints. MW designed and implemented the extensions to previous constraint‐layout and connector routing algorithms, and advised about constraint specification. KM and FS supervised the project and contributed to the intellectual design of the described techniques, KM primarily on the layout side, and FS predominantly on the biology and translation side. All authors contributed to writing the article. All authors read and approved the final manuscript.
